# Studying the role of fascin-1 in mechanically stressed podocytes

**DOI:** 10.1038/s41598-017-10116-4

**Published:** 2017-08-30

**Authors:** Felix Kliewe, Christian Scharf, Henrik Rogge, Katrin Darm, Maja T. Lindenmeyer, Kerstin Amann, Clemens D. Cohen, Karlhans Endlich, Nicole Endlich

**Affiliations:** 1 Department of Anatomy and Cell Biology, University Medicine Greifswald, Greifswald, Germany; 2 Department of Ear, Nose and Throat Diseases, University Medicine Greifswald, Greifswald, Germany; 3 Nephrological Center, Medical Clinic and Policlinic IV, University of Munich, Munich, Germany; 4 Department of Nephropathology, University Medicine Erlangen, Erlangen, Germany

## Abstract

Glomerular hypertension causes glomerulosclerosis via the loss of podocytes, which are challenged by increased mechanical load. We have demonstrated that podocytes are mechanosensitive. However, the response of podocytes to mechanical stretching remains incompletely understood. Here we demonstrate that the actin-bundling protein fascin-1 plays an important role in podocytes that are exposed to mechanical stress. Immunofluorescence staining revealed colocalization of fascin-1 and nephrin in mouse kidney sections. In cultured mouse podocytes fascin-1 was localized along actin fibers and filopodia in stretched and unstretched podocytes. The mRNA and protein levels of fascin-1 were not affected by mechanical stress. By Western blot and 2D-gelelectrophoresis we observed that phospho-fascin-1 was significantly downregulated after mechanical stretching. It is known that phosphorylation at serine 39 (S39) regulates the bundling activity of fascin-1, e.g. required for filopodia formation. Podocytes expressing wild type GFP-fascin-1 and non-phosphorylatable GFP-fascin-1-S39A showed marked filopodia formation, being absent in podocytes expressing phosphomimetic GFP-fascin-1-S39D. Finally, the immunofluorescence signal of phosphorylated fascin-1 was strongly reduced in glomeruli of patients with diabetic nephropathy compared to healthy controls. In summary, mechanical stress dephosphorylates fascin-1 in podocytes *in vitro* and *in vivo* thereby fascin-1 may play an important role in the adaptation of podocytes to mechanical forces.

## Introduction

Podocytes, terminally differentiated cells in the glomerulus, are attached to the glomerular basement membrane (GBM) by their foot processes and interdigitate in a zipper-like fashion. The interdigitating foot processes are connected by the slit diaphragm that is formed by homophilic interaction of the transmembrane protein nephrin. This complex 3D morphology is stabilized by the actin cytoskeleton which is associated via linker and transmembrane proteins also with the extracellular matrix. Alterations of the actin cytoskeleton or actin-associated proteins often result in the effacement of foot processes or in the detachment of podocytes. Both changes cause the loss of high-molecular weight proteins through the filtration barrier (proteinuria) and may eventually progress to end stage renal disease^1–4^. Since mice and rats suffering from hypertension or diabetic nephropathy often develop glomerular hypertension, it is suggested that this could be the reason for the podocyte damage and the loss of podocytes in patients with hypertension and diabetic nephropathy (DN)^5^.

To investigate whether podocytes are mechanosensitive, our group cultured mouse podocytes on flexible silicone membranes and exposed them to mechanical stress for three days. We discovered that podocytes completely reorganize their actin cytoskeleton in response to mechanical stress^6^. Instead of transversal stress fibers, actin filaments were organized radially converging into an actin-rich center (ARC). Moreover, we observed that stretched podocytes developed more thin and more extended protrusions compared to unstretched podocytes^6^.

Until today it is still unknown which pathway is required to induce the formation of podocyte foot processes *in vivo* and *in vitro*. However, it has already been shown that the podocyte-specific loss of the small Rho GTPase Cdc42, which is responsible for the formation of filopodia, resulted in a severe kidney phenotype with effacement of foot processes^7, 8^. Here, we studied the role of another protein that is essential for the formation of filopodia and cell protrusions, fascin-1. It is a 55 kDa monomeric actin-binding protein that is highly expressed in neurons and dendritic cells^9–13^. Furthermore, in most invasive cancers, fascin-1 is highly upregulated and the expression can be used as a prognostic marker^14–16^. Fascin-1 contains two actin binding sides, and it was shown that its bundling activity is regulated via phosphorylation of serine-39 by PKC^11, 17, 18^. Interestingly, Villari and colleges have recently shown that fascin-1 can directly interact with microtubules in an actin-binding independent way, which influences the stability of focal adhesions and the dynamics of cells^19^.

In the present study, we demonstrate that fascin-1 is expressed in podocytes and that it is regulated by mechanical stress. We show that adaptation of podocytes to mechanical stress is supported by dephosphorylation of fascin-1. Since we provide evidence that phosphorylation of fascin-1 is altered in DN, regulation of the bundling activity of fascin-1 may play an important role in glomerular disease.

## Results

### Fascin-1 is specifically expressed in podocytes of mice

**Figure 1:**
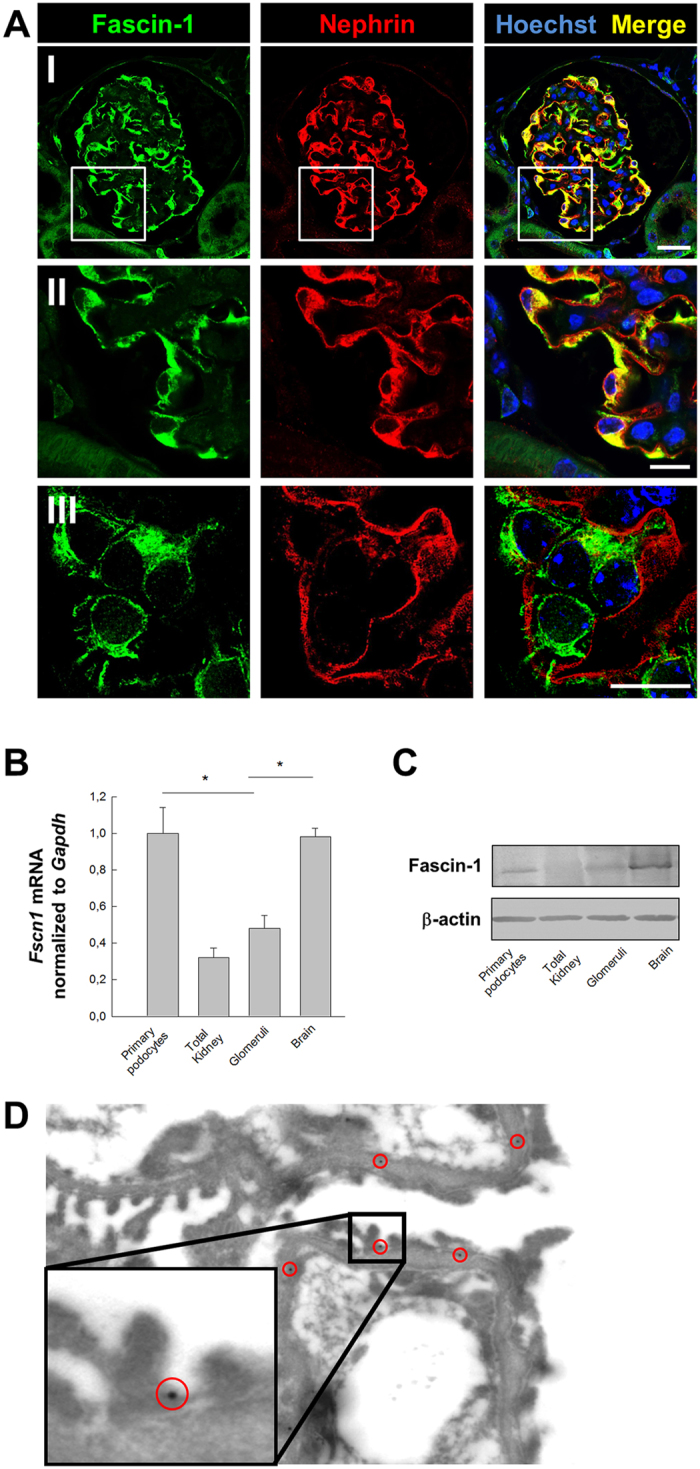
Fascin-1 is predominantly expressed in mouse podocytes *in vivo*. (**A**) Kidney sections were stained for fascin-1 (green) and nephrin (red) (I,II). By superresolution microscopy SIM, single podocytes were visualized expressing fascin-1 in their major processes (III, green). Nuclei were stained with Hoechst (blue). Scale bars represent 25 µm (I) and 10 µm (II, III), respectively. (**B**) Quantitative measurement of fascin-1 mRNA isolated of primary podocytes, total kidney, glomeruli and brain as a control. (**C**) Western blot of the expression of fascin-1 isolated from total kidney, glomeruli, primary podocytes and brain (as a positive control). Values were normalized with Gapdh for qRT-PCR and β-actin for Western blot analysis. Data are presented as means ± SEM; *p < 0.05. (**D**) Immunoelectron microscopy reveals the localization of fascin-1 also in foot processes near the slit diaphragm. The magnification is 40,000x.

To determine the localization of fascin-1 in the glomerulus, histological sections of mouse kidneys were stained with antibodies against fascin-1 and the podocyte-specific protein nephrin. Figure 1A shows that fascin-1 is specifically expressed in podocytes and is colocalized with nephrin indicated by the yellow staining in the merged picture (Fig. 1A I, II). Moreover, by structured illumination microscopy (SIM), a super resolution microscope technology, we found that fascin-1 is especially expressed in the cell body and processes of podocytes *in vivo* (Fig. 1A III). The expression of fascin-1 in primary podocytes, whole kidney, glomeruli and brain was verified by qRT-PCR and Western blot (Fig. 1B,C). Isolated mouse glomeruli and primary podocytes (PP) showed a strong expression of fascin-1 mRNA and protein. Immunoelectron microscopy revealed that fascin-1 is also located in podocyte foot processes, sometimes near the slit diaphragm. Furthermore, fascin-1 was faintly expressed in the endothelium (Fig. 1D). In contrast, the negative control (staining only with the gold-labeled secondary antibody) showed no signal in the podocyte (Supplementary Fig. 1).

### Mechanical stretch does not affect the expression of fascin-1

**Figure 2:**
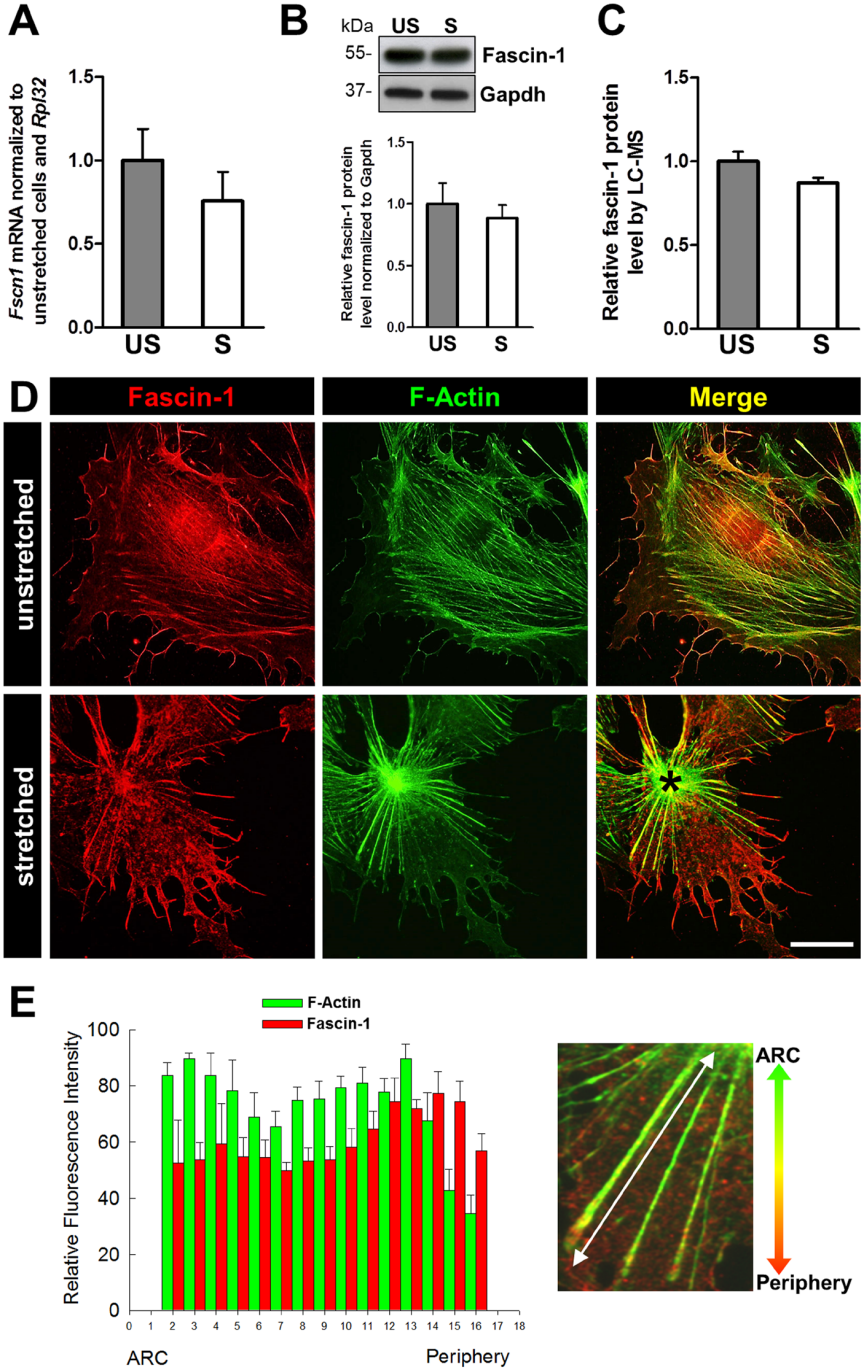
Fascin-1 expression in response to mechanical stretch. (**A**) Quantitative RT-PCR revealed no significant differences in fascin-1 mRNA expression in S/US podocytes (n = 3). (**B**) Western blot showed no difference in the expression for fascin-1 in S/US podocytes. Relative fascin-1 protein levels on Western blots were quantified for unstretched (grey bar) to stretched cells (white bar) and revealed only a slightly but not significant down-regulation of fascin-1 after mechanical stress (n = 5). (**C**) LC-MS analysis of fascin-1 protein level (n = 4). (**D**) Immunofluorescence colocalization analysis of fascin-1 (red) and F-actin (green). Unstretched podocytes showed transversal actin fibers which reorganized into radial actin fibers converging into an ARC due to mechanical stress. In stretched podocytes the fluorescence intensity of the actin staining decreases anterogradely starting from the centre, whereas the intensity of the fascin-1 staining increases as depicted in the merged picture. (**E**) The quantitative analysis of the fluorescence intensities of stretched filaments is shown in the histogram. For quantification, the fluorescence intensities of F-actin and fascin-1 along single stress fibers (n = 5) were measured in 15 representative stretched cells starting from the actin rich-center (ARC) to the periphery of the cell. Experiments were normalized to *Rpl32* mRNA (**A**) or Gapdh protein levels (**B**,**C**). Stretch parameters: cycle frequency of 0.5 Hz, linear strain of 5% for 3 days. Data are presented as means ± SEM. Scale bars represent 25 µm.

To find out whether fascin-1 is regulated in cultured podocytes by mechanical stretch, we determined the expression of fascin-1 in stretched (S) and unstretched (US) podocytes by qRT-PCR, Western blot and LC-MS analysis (Fig. 2A-C). We found a slight but nonsignificant decrease of the fascin-1 mRNA expression down to 75 ± 17% induced by mechanical stretch (Fig. 2A). The Western blot also showed that the amount of fascin-1 remained unchanged during mechanical stretch (S/US ratio: 88 ± 11%) (Fig. 2B). To validate this finding, an LC-MS analysis revealed that the expression of fascin-1 was not significantly regulated by mechanical stress (S/US ratio: 87 ± 3%, n = 4, *p* = 0.37) (Fig. 2C).

### Fascin-1 is localized along actin fibers and in filopodia of stretched and unstretched podocytes

In cultured podocytes, fascin-1 is localized along actin fibers, filopodia, and focal adhesions as shown in Fig. 2D. To find out whether mechanical stretch affects the localization of fascin-1, we stretched podocytes that were cultured on flexible silicone membranes, for 3 days with 0.5 Hz and 5% elongation as described previously^6^. Studying the mechanical stretch-induced reorganization of the actin cytoskeleton from transversal stress fibers into radial stress fibers revealed that fascin-1 remained associated with actin fibers as well as focal adhesions (Fig. 2D). An accumulation of fascin-1 in the actin-rich center (ARC), where the radial stress fibers converge, was not found (asterisk in Fig. 2D). In US podocytes fascin-1 intensity was higher towards the ends than in the middle of stress fibers. Likewise, measurement of the fluorescence intensity along single radial stress fibers in S podocytes showed an increase of the expression of fascin-1 from the ARC towards the periphery (Fig. 2E).

### The knockdown of fascin-1 influences the morphology, the adhesion and the number of focal adhesions of stretched podocytes

**Figure 3:**
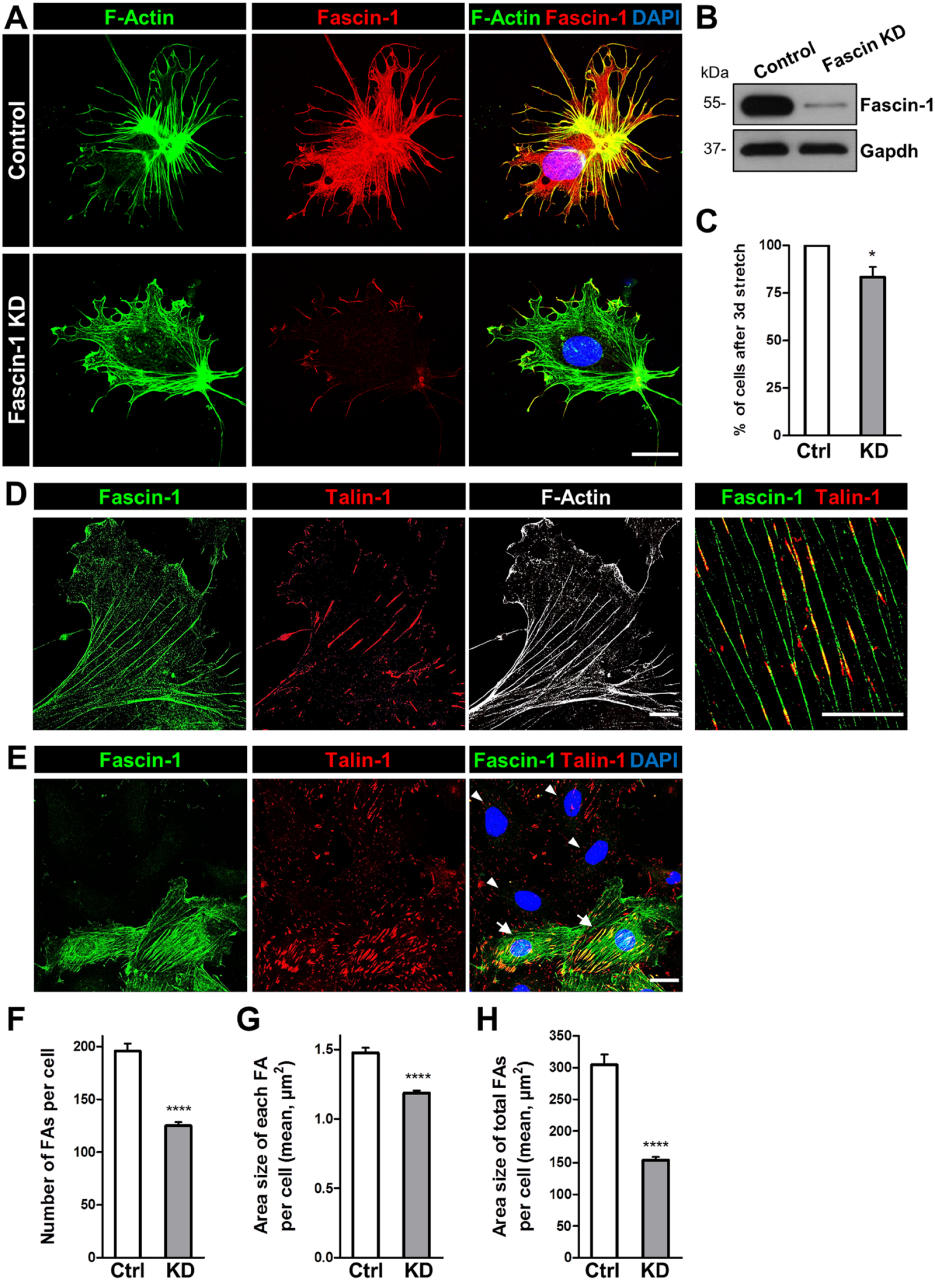
Knockdown of fascin-1 influences the morphology of stretched podocytes and reduces the number of focal adhesions. (**A**) Immunofluorescence staining of fascin-1 (red) and F-actin (green) of control and fascin-1 KD cells after 3 days of biaxial mechanical stress with 0.5 Hz and 5% elongation. (**B**) Verification of fascin-1 KD by Western blot. (**C**) Podocytes adhesion after 3 days of stretch (n = 3). (**D**) Structured illumination microscopy (SIM) images showed co-localization of fascin-1 (green), talin-1 (red) and F-actin (white) in cultured podocytes. (**E**) Immunofluorescence staining of fascin-1 (green) and talin-1 (red) of control and fascin-1 KD cells. Fascin-1 KD (cells marked with arrowheads) reduced the number of talin 1-positive focal adhesions. Control podocytes (marked by arrows) showed more and thicker talin-1 contacts. (F-H) Quantitative analysis of focal adhesions (talin-1) in control (n = 185) and fascin-1 KD cells (n = 280). (**F**) Number of focal adhesions (FAs) per cell was reduced by 36 ± 2% after the KD of fascin-1 (125 ± 3 focal contacts per cell) compared to control transfected podocytes (196 ± 7 focal contacts per cell), *p* = 2.14E-22. (**G**) Area size of each FA per cell was reduced by 19.6 ± 1.1 % after fascin-1 KD (Ctrl: 1.48 ± 0.04 µm^2^, KD: 1.19 ± 0.02 µm^2^, *p* = 1.95E-14). (**H**) Area size of total FAs per cell showed a reduction by 49.5 ± 1.6% in fascin-1 KD podocytes (Ctrl: 304 ± 16 µm^2^, KD: 154 ± 5 µm^2^, *p* = 1.72E-22). Error bars indicate SEM; *p < 0.05; ****p ≤ 0.0001. Scale bars represent 25 µm (**A**,**E**) and 10 µm (**D**), respectively.

To study the role of fascin-1 in cultured podocytes that were exposed to mechanical stress, we knocked down (KD) fascin-1 by specific siRNA. For the validation of the KD of fascin-1, immunocytochemistry and Western blots were performed (Fig. 3A,B). As shown in Fig. 3A, the loss of fascin-1 was accompanied by a reduction of thin protrusions and filopodia (Fig. 3A). However, the KD of fascin-1 did not disturb the reorganization of the F-actin cytoskeleton including the formation of ARCs induced by mechanical stretch. After 3 day mechanical stretch, more than 17 ± 5% of fascin-1 KD podocytes were lost compared to stretched control cells, suggesting an important role of fascin-1 for the attachment of podocytes (Fig. 3C).

Using SIM we found that fascin-1 and talin-1 co-localize at focal adhesions in cultured podocytes (Fig. 3D). As shown in Fig. 3E, the fascin-1 KD influenced the size and the number of focal adhesions significantly (arrowheads). By the quantification of more than 450 podocytes (n = 3 experiments), we found that the number of focal contacts was reduced by 36 ± 2% after the KD of fascin-1 compared to control transfected podocytes (ctrl: 196 ± 7 focal contacts per cell, fascin-1 KD: 125 ± 3 focal contacts per cell, *p* = 2.14E-22, Fig. 3F).

Furthermore, the area size of each focal contact per cell decreased from 1.48 ± 0.04 µm^2^ in control cells to 1.19 ± 0.02 µm^2^ in fascin-1 KD cells (Fig. 3G). Total focal adhesion area per cell was reduced by 49.5 ± 1.6% in fascin-1 KD podocytes compared to the control (Fig. 3H).

### Mechanical stretch induces dephosphorylation of fascin-1 at Ser-39

Since the bundling capacity of fascin-1 is dependent on the phosphorylation of Ser-39, we studied whether phosphorylation of fascin-1 is regulated by mechanical stretch. After isolation of phospho-proteins from S and US podocytes, we performed Western blots that were stained with an antibody against fascin-1. Protein load and the efficiency of the phospho-protein isolation were checked by stain-free imaging (Supplementary Fig. 2A) and Western blot (Supplementary Fig. 2B).

**Figure 4:**
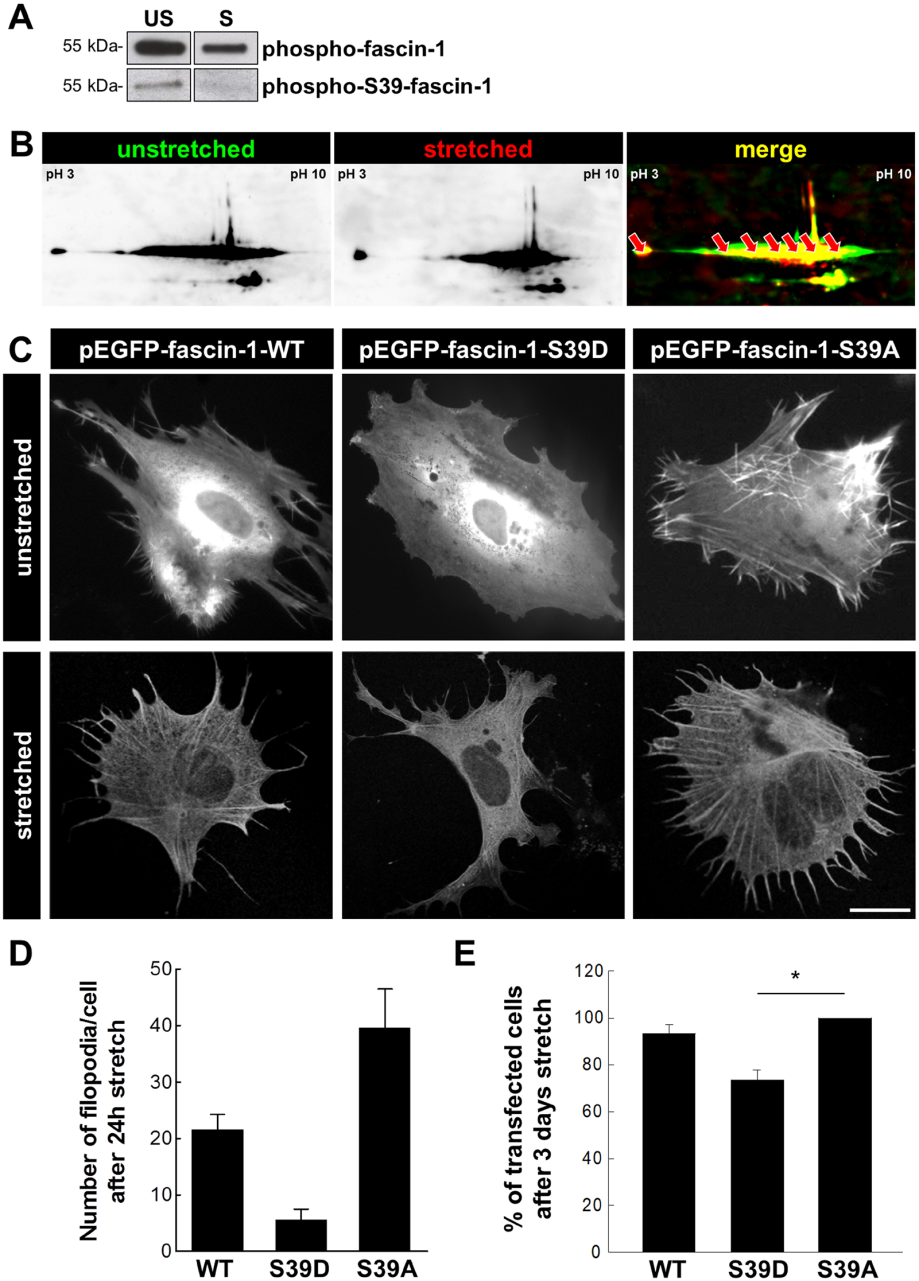
Phosphorylation of fascin-1 inhibits the formation of filopodia and phospho-S39-fascin-1 is dephosphorylated by mechanical stretch. (**A**) After isolation of phospho-proteins from S/US podocytes, a Western blot was performed. After staining for fascin-1 and phospho-S39-fascin-1, respectively, only a weak band was detected in stretched podocytes compared to unstretched podocytes. Blots of representative experiments of five independent experiments are shown. Protein load was checked by stain-free imaging. (**B**) 2-D gel image of S/US podocyte protein extract, which detects a shift of the fascin-1 protein spots to the alkaline pH-range (n = 3). The different fascin-1 spots (marked by arrowheads) were determined by LC-MS. (**C**) Podocytes were transfected with a plasmid encoding for wildtype-, non-phosphorylatable mutant eGFP-fascin-1-S39A (S39A) and the phosphomimetic mutant eGFP-fascin-1-S39D (S39D). Compared to wildtype and S39A-fascin-1, S39D-expressing podocytes were unable to develop filopodia neither under unstretched nor under stretched conditions. (**D**) Number of filopodia in wildtype-, S39A- and S39D-fascin-1 transfected cells after mechanical stress for 24 h. Error bars indicates SEM. (**E**) After transfection with a plasmid for eGFP-fascin-1-S39A, number of adherent cells after 3 days of mechanical stress is significantly higher than after transfection with eGFP-fascin-1-S39D. Bars with SEM; *p < 0.05; n = 3.

As shown in Fig. 4A the signal for phosphorylated fascin-1 was significantly reduced due to mechanical stretch (n = 5). By the use of a specific antibody we identified that fascin-1 is dephosphorylated specifically on Ser-39 under mechanical stretch (Fig. 4A).

By 2-D electrophoresis, we identified more than the two expected spots for non- and phosphorylated S39-fascin-1 in US podocytes. As shown in Fig. 4B, five additional spots (analysed and verified by LC-MS) were detected indicating that fascin-1 has several residues that can be phosphorylated. After mechanical stretch, we observed a shift of the position of fascin-1 spots from the low pH- to the alkaline pH-range as expected after dephosphorylation of fascin-1 due to mechanical forces.

Since filopodia formation is dependent on the serine phosphorylation of fascin-1 at position 39 (Ser-39)^11, 17, 20^, we wanted to find out whether the generation of filopodia in podocytes depends on the phosphorylation of Ser-39 in S and US podocytes as well. For this purpose, we transfected podocytes with different plasmids encoding for two fascin-1 mutants: the non-phosphorylatable mutant eGFP-fascin-1-S39A (S39A) and the phosophomimetic mutant eGFP-fascin-1-S39D (S39D).

US podocytes that were transfected with the wildtype fascin-1 (WT-fascin-1) and S39A plasmid spontaneously developed a large number of highly dynamic filopodia as shown by time lapse microscopy (Supplementary movie 1). In contrast, S39D-expressing podocytes were unable to develop filopodia neither under US nor S conditions (Fig. 4C,D). Furthermore, we studied whether the phosphorylation of fascin-1 on Ser-39 has an influence on the adhesion of podocytes under mechanical stretch. We found that significantly less podocytes, expressing the phosphomimetic form of fascin-1, were attached after 3 days mechanical stretch compared to podocytes expressing S39A-fascin-1 (−27 ± 4%, *p* < 0.05; n = 3, Fig. 4E).

### Cell spreading and adhesion of podocytes is related to the expression of fascin-1 under mechanical forces

**Figure 5:**
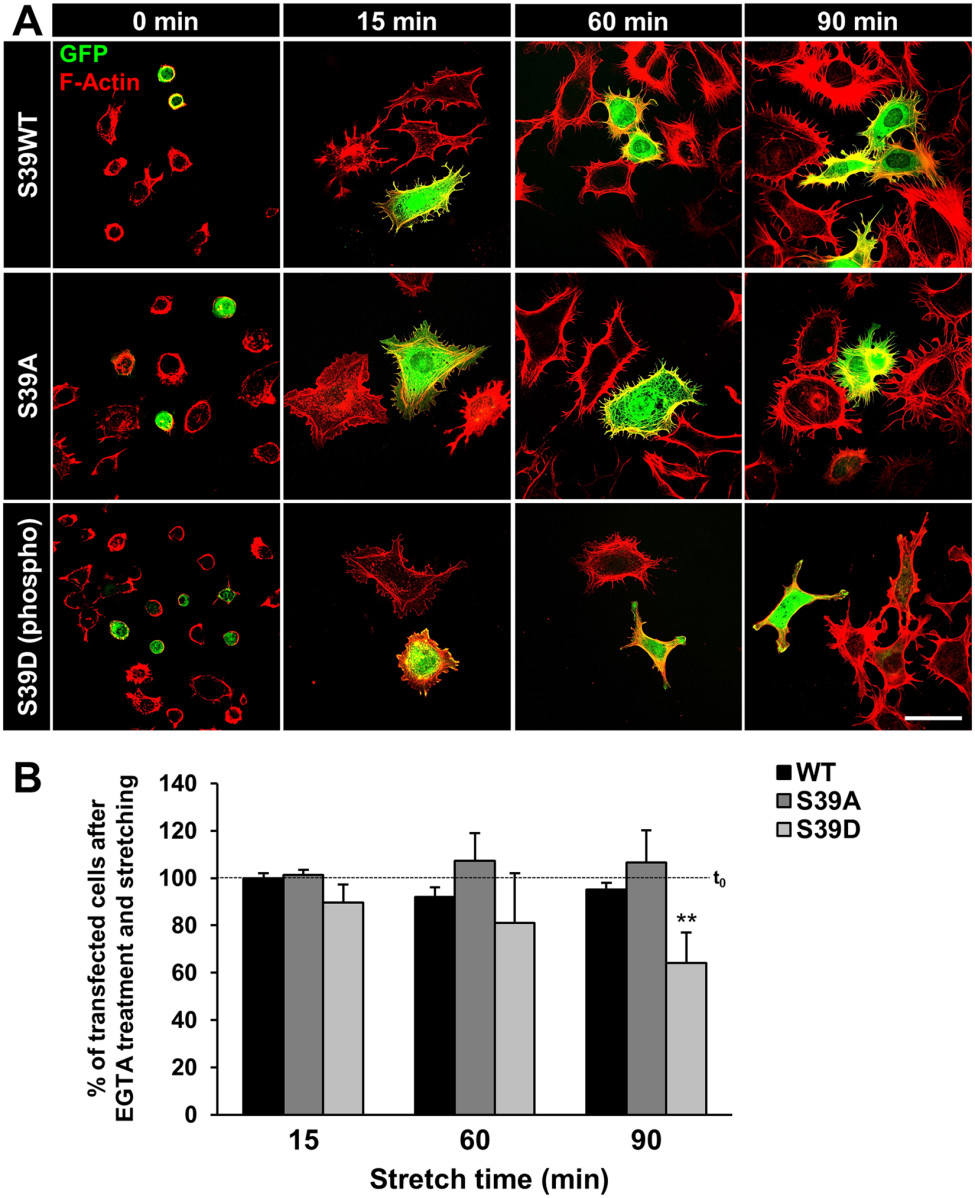
Effect of S39A-fascin-1, S39D-fascin-1 and wildtype fascin-1 on cell spreading and filopodia formation during mechanical stress. (**A**) Confluent podocytes were transfected with eGFP-fascin-1 wildtype (WT), eGFP-fascin-1-S39A and eGFP-fascin-1-S39D and Ca^2+^-depleted for 30 min. After re-addition of Ca^2+^, the cells were mechanical stretched. Cell spreading and filopodia formation occurred within 60 min and was completed after 90 min in control cells as well as in podocytes expressing eGFP-fascin-1-S39A. In contrast, eGFP-fascin-1-S39D transfected podocytes showed a poor spreading and no filopodia formation during 90 min. Scale bar represent 50 µm. (**B**) Quantitative analysis showed that 95 ± 3% of wildtype cells and 106 ± 14% of S39A-fascin-1 transfected cells were still adherent after 90 min in the presence of mechanical stress. In contrast, the number of S39D-fascin-1 transfected podocytes significantly decreased to 64 ± 13%. Data are mean ± SD of n = 4 experiments, **p < 0.01.

To further evaluate the role of fascin-1 for cell adhesion, podocytes were transfected with a plasmid encoding for S39A-fascin-1, S39D-fascin-1 and WT, respectively, followed by calcium depletion combined with mechanical stretch. Calcium depletion by EGTA induced a rounded morphology of the podocytes independent of the phosphorylation at Ser-39. After re-addition of calcium, we analysed the cell spreading and the formation of filopodia in S39A-, S39D- and in WT-fascin-1 transfected podocytes at different time points (15, 60 and 90 min). Podocytes expressing S39D-fascin-1 showed only a limited spreading and no formation of filopodia in contrast to S39A-fascin-1 and WT-fascin-1 expressing podocytes (Fig. 5A). After 60 min exposure to mechanical stretch, S39A-fascin-1 and WT-fascin-1 expressing podocytes were fully spread and after 90 min cell-cell contacts were reestablished. Quantitative analysis revealed that the number of attached S39A-fascin-1 and WT-fascin-1 transfected podocytes was not significantly changed after 90 min (106 ± 14% and 95 ± 3%, respectively). In contrast, the number of attached S39D-fascin-1 transfected podocytes was significantly decreased to 64 ± 13% after 90 min stretch (p < 0.01, n = 4, Fig. 5B).

### Patients suffering from diabetic nephropathy have a reduced phospho-S39-fascin-1 expression in podocytes

**Figure 6:**
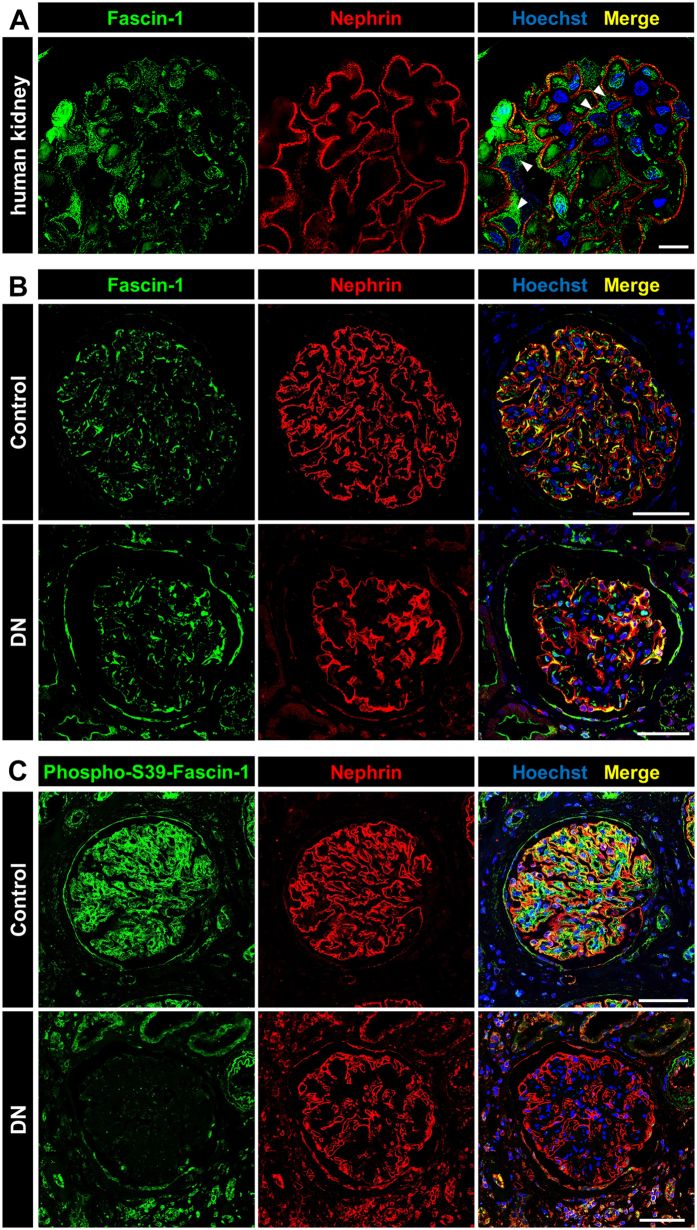
Kidney biopsies of patients suffering from diabetic nephropathy (DN) have a reduced phospho-S39-fascin-1 expression. (**A**) Expression of fascin-1 (green) and nephrin (red) in human kidneys taken by SIM. Podocytes ﻿are marked with arrowhea﻿d﻿﻿s. (**B**) Expression of fascin-1 is not reduced by patients suffering from DN. (**C**) Expression of phospho-S39-fascin-1 was reduced in glomeruli of patients suffering from DN compared to control tissue. Scale bars represent 10 µm (**A**) and 50 µm (**B**,**C**), respectively.

To find out whether fascin-1 is also expressed in human glomeruli, histological sections of kidney biopsies were stained for fascin-1 as well as for the podocyte-specific protein nephrin and analyzed by SIM. Figure 6A shows that fascin-1 is expressed in human podocytes (marked by arrowheads).

To determine whether fascin-1 is regulated in patients suffering from diabetic nephropathy (DN), we assessed the mRNA expression level of fascin-1 in microdissected glomeruli from renal biopsies of patients suffering from DN (n = 14) and compared them with healthy living donors (n = 8). Patients with DN displayed no significant change in fascin-1 expression compared to controls (fold change: 1.14, *p* = 0.664; Mann-Whitney *U* test). This is in agreement with the results obtained by the staining of human biopsies, which showed no alteration in the fascin-1 expression in podocytes of control and DN biopsies in three different patients (Fig. 6B). Interestingly, the expression of fascin-1 increased in parietal epithelial cells and proximal tubule cells in patients with DN. Strikingly, immunostaining of kidney sections with a specific antibody against phosphorylated fascin-1 at serine 39 revealed that phospho-S39-fascin-1 is only present in healthy glomeruli, but not in glomeruli of patients suffering from DN (Fig. 6C). This significant decrease of the phospho-S39-fascin-1 expression was specifically seen in all glomeruli of three independent DN biopsies. Interestingly, the expression of phospho-S39-fascin-1 seems not to be affected in tubule cells.

## Discussion

Kidney function is highly dependent on the morphology of podocytes. The loss of their complex arborized cell morphology and the disappearance of the interdigitating, thin membrane protrusions, the foot processes, result in a leakage of the filtration barrier. Until today it is still unsolved how these protrusions develop and how the loss of the foot process morphology, namely effacement, could be inhibited or even reversed. Fascin-1, an actin-bundling protein, has already been shown to be essential for the formation of filopodia and to control the arborization of neurons^9–11^. This is very interesting since podocytes and neurons share some similarities^21–24^. Beside this it was demonstrated that fascin-1 plays an important role for cell adhesion and migration of neurons and other cell types^19^.

In the present study, we show that podocytes express fascin-1 *in vivo* and *in vitro*. The main interest of our study was focused on the role of fascin-1 in response to mechanical stretch. It is well known that diabetic nephropathy is often associated with glomerular hypertension, which leads to severe damage and loss of podocytes^5^. Recently, we have found that podocytes are mechanosensitive and respond to mechanical stretch by the generation of long and thin cell protrusions, by the reorganization of the actin cytoskeleton as well as by detachment^25^. However, it is poorly understood which proteins are responsible for such a reorganization or detachment induced by mechanical stretch *in vitro* as well as *in vivo*. Therefore, our study wanted to clarify whether the bundling protein fascin-1 is essential for the stability and morphology of mechanically stretched podocytes.

Surprisingly, we did not find any difference in the protein and mRNA expression of fascin-1 in S compared to US podocytes. However, proteome analysis of S and US podocytes by 2D gel electrophoresis revealed significant differences in the intensity and position of the identified fascin-1 spots, reflecting the phosphorylation status of fascin-1. Ono and coworkers showed that the phosphorylation/dephosphorylation of Ser-39 is crucial for the actin bundling activity of fascin-1^11, 26^. Furthermore, it was reported by different researchers that the formation of filopodia in mouse melanoma and neuroblastoma cells was completely inhibited by the phosphorylation of Ser-39^11, 20^. By the use of a specific antibody for Ser-39-phosporylated fascin-1, we showed that mechanical stretch induces a nearly complete dephosphorylation of fascin-1 at Ser-39. This suggests that actin filaments are tightly bundled by the small protein fascin-1 in response to mechanical stretch. Such changes of filament bundling could influence the traction forces of podocytes, as it was reported for fibroblasts and cancer cells by Elkhatib and colleagues^27^. They found that fascin-1 reduced the contractility of actin filaments by inhibiting access of myosin II and increased the actin polymerization rate at stress fiber termini in fibroblasts and cancer cells. We and other groups have shown that the actin polymerization at focal adhesions is dependent on myosin II and may be important for the force transmission at focal adhesions to an extracellular matrix^25, 28, 29^. Therefore, we speculated that fascin-1 might be essential for podocytes adhesion especially under mechanical stretch. After the knockdown of fascin-1 in cultured podocytes by siRNA, podocytes failed to develop filopodia and extended protrusions, which is in agreement with the results observed for neurons and cancer cells^11^. Furthermore, we observed a significant detachment of stretched podocytes after fascin-1 knockdown that might be caused by the loss of focal adhesions due to reduced fascin-1 levels.

We identified seven differently charged fascin-1 spots in the proteome analysis, suggesting that Ser-39 is not the only phosphorylation site of fascin-1. Interestingly, the analysis revealed that fascin-1 possesses a disproportionally high number of phosphorylatable amino acids like serine, tyrosine and threonine. With one exception, i.e. Ser-274, the function of phosphorylation at these residues is unknown. Villari *et al*. have recently shown that phosphorylation at Ser-274 is important for the binding of fascin-1 to microtubules which contributes to a fascin-dependent control of focal adhesion dynamics and cell migration^19^. This sheds light on a new role for fascin-1, which might have implications also for podocytes where microtubuli are only localized in major processes and actin mainly in foot processes *in vivo*. One may speculate that fascin-1 could play a role as a linker between both cytoskeletal proteins.

As mentioned before, fascin-1 is not only responsible for the bundling of actin to tight filaments but also plays a central role in cell adhesion and migration, which has already been demonstrated in cancer cells as well as fibroblasts^30^. In this regard, Johnson and colleagues have recently shown that F-actin bundles and filopodia containing fascin-1 serve as templates for the formation and orientation of lamellipodia through adhesion-based signalling^30^. We found that the transfection of cultured podocytes with the phosphomimetic S39D mutant of fascin-1 led to the loss of filopodia and diminished cell spreading compared to podocytes expressing the non-phosphorylatable S39A mutant. This might be caused by the inability of S39D-expressing podocytes to form such thin, actin-containing protrusions, which act as guides for cell spreading. Recently, we have made a similar observation with CD151-overexpressing podocytes. The upregulation of the tetraspanin CD151 significantly increased the generation of thin arborized cell protrusions (TAPs) that served as rails for cell spreading after the recovery from calcium depletion^31^.

In our study we have seen that fascin-1 is essential for the formation of thin protrusions as well as for the adhesion of cultured podocytes exposed to mechanical stretch. To prove whether this could be of clinical relevance, we studied the expression of fascin-1 and the Ser-39 phosphorylation status of fascin-1 in kidney biopsies of patients suffering from DN. Since DN is often associated with glomerular hypertension, which leads to a higher mechanical stress of podocytes *in vivo* due to an increased wall tension of the glomerular capillaries^5^, we hypothesized that Ser-39 could also be dephosphorylated in patients with DN. Indeed, while there was no difference in the expression of fascin-1 in patients with DN compared to controls, and while fascin-1 was phosphorylated at Ser-39 in glomeruli of control biopsies, fascin-1 was almost completely dephosphorylated at Ser-39 in glomeruli of patients suffering from DN. This finding suggests that the stability of podocytes *in vivo* and *in vitro* might be supported by fascin-1 that organizes actin into tight filament bundles to withstand increased mechanical stress, like it is observed in many patients suffering from DN.

Taken together, our study shows that fascin-1 plays an essential role for the morphology and function of mechanically stretched podocytes. Interestingly, mechanical stress does not regulate fascin-1 at the mRNA or protein expression level in podocytes, but through the phosphorylation/dephosphorylation at Ser-39.

## Methods

### Cell culture

Conditionally immortalized podocytes (SVI; CLS Cell Line Service GmbH, Eppelheim, Germany) were handled as described previously^6^.

### Mouse experiments

For the isolation of kidneys, glomeruli and primary podocytes, mice (C57BL/6) were anaesthetized and sacrificed. All procedures with animals were performed in accordance with the national animal protection guidelines which is conform to the National Institutes of Health Guide for the Care and Use of Laboratory Animals and were approved by the local governmental authorities (LALFF (Landesamt für Landwirtschaft, Lebensmittelsicherheit und Fischerei Mecklenburg-Vorpommern)). Glomeruli and primary podocytes were isolated by the sieving technique that was described previously^6^.

### Mechanical stress experiments

Mechanical stress experiments were performed according to our previous study^6^. Differentiated podocytes were cultured for 3 days in six-well plates with a flexible silicone bottom (Bioflex, Flexcell^®^ International Corporation, Burlington, NC, USA). Plates were mounted on a manifold connected to the stretch apparatus (CLS Cell Line Service GmbH). Cycle frequency was 0.5 Hz and maximum linear strain 5%. Number of adherent cells was determined by counting stained podocytes in 40 microscopic fields.

### RNA extraction, cDNA synthesis and qRT-PCR

Samples from cells/tissues were processed in Tri-Reagent (Sigma-Aldrich, St. Louis, MO, USA) according manufacturer’s instructions. For cDNA synthesis, 1 µg of the isolated total RNA was transcribed using the QuantiTect Reverse Transcription Kit (Qiagen, Hilden, Germany). The quantitative Real-time PCR (qRT-PCR) analysis was performed on a LightCycler Nano (Roche Diagnostics GmbH, Mannheim, Germany) using the iTaq Universal SYBR Green Supermix (Bio-Rad) in presence of specific primers for *Fscn1* (sense: 5′-AACCCCTTGCCTTTCAAACT-3′; antisense: 5′-CAGAGTTCCCCATGGAAAGA-3′), *Gapdh* (sense: 5′-ACCCAGAAGACTGTGGATGG-3′; antisense: 5′-CACATTGGGGGTAGGAACAC-3′) and *Rpl32* (sense: 5′-AGTTCATCAGGCACCAGTCAG-3′; antisense: 5′-ATCAGGATCTGGCCCTTGAAC-3′). Relative expression was calculated by normalizing values to *Gapdh* or *Rpl32*.

### Western blot

Cells were trypsinized, washed twice in PBS and dissolved in lysis buffer containing 50 mM octylglucoside, 50 mM Tris, 150 mM NaCl, 10 mM CaCl_2_, 1 mM MgCl_2_ and 1.5 mg/ml complete protease inhibitor (Roche Diagnostics). Protein extracts were subjected to Western blot analysis or phosphopeptide isolation using Pierce™ Phosphoprotein Enrichment Kit (Thermo Fisher Scientific). After addition of SDS-PAGE sample buffer (final concentrations: 32 mM Tris-HCl, 1% SDS, 5% glycerol, 0.05% bromphenol blue, 3.25% 2-mercaptoethanol, pH 6.8), the cell lysates were heated at 95 °C, 5 min. Lysate aliquots containing 2 µg of phosphoproteins and 20 µg of total proteins were separated using a 4–20% Mini-PROTEAN^®^ TGX™ Gel, (Bio-Rad Laboratories) and transferred to a nitrocellulose membrane. Membranes were immersed overnight in blocking buffer (10 mM Tris, 100 mM NaCl, 5% non-fat dry milk, 0.2% Tween-20, pH 7.5) and incubated for 2 h at room temperature in TBS-Tween (0.2%) with the following antibodies: anti-fascin-1 (sc-21743, Santa Cruz), anti-fascin-1 Ser-39-phospho-specific antibody (FP2661; ECM Biosciences, Versailles, KY, USA) and anti-Gapdh (sc-25778, Santa Cruz). Blots were incubated for 1 h with HRP–conjugated secondary antibody anti**-**mouse (sc-2031, Santa Cruz) or anti**-**rabbit (sc-2030, Santa Cruz) and developed using SuperSignal™ West Pico Chemiluminescent Substrate (Thermo Fisher Scientific) and finally exposed to X-ray films (Fujifilm Super RX, FUJIFILM, Tokyo, Japan). In some cases detection of bound antibodies was done with alkaline phosphatase-labelled secondary antibody (anti-mouse AP, Acris Antibodies GmbH, Herford, Germany) and phosphatase conjugate substrate-kit (Bio-Rad Laboratories). For the relative quantification, developed x-ray films were scanned and analyzed using ImageJ (version 1.48 v, NIH, Bethesda, MD, USA)^32^. Mean grey values of specific fascin-1 signals were determined and normalized to mean grey values of Gapdh signals.

### 2-D gel electrophoresis

Stretched respectively unstretched cells were harvested and proteins were extracted in lysis buffer containing 8 M urea and 2 M thiourea. Protein content was determined by the Bradford method^33^. 2-DE was performed as described^34^. Briefly, 125 µl protein solution (containing 40 µg protein) was suspended in rehydration medium consisting of 8 M urea, 2 M thiourea, 2% (w/v) CHAPS, 0.5% (v/v) IPG buffer pH 3–10, and a few grains of bromophenol blue in a final volume of 350 µl. The samples were applied to 7 cm pH 3–10 non-linear gradient IEF gel strips for isoelectric focussing (IPGphor apparatus, GE Healthcare, Little Chalfont, UK). The IEF gel strips were allowed to reswell for 1 min at 200 V. Then 3500 V were applied for 2.5 h. Gel strips were equilibrated for 15 min each in an SDS equilibration buffer consisting of 50 mM Tris-HCl, pH 8.8, 6 M urea, 30% (v/v) glycerol, 2% (w/v) SDS, a few grains of bromophenol blue, and 1% (w/v) dithiothreitol or 2.5% (w/v) iodoacetamide, respectively. The second dimension separation was performed using 12.5% polyacrylamide gels in the presence of 0.1% (w/v) sodium dodecylsulfate. Gels were run at 20 mA for 10 min and 40 mA for about 1.5 h in vertical electrophoresis apparatus (Bio-Rad Laboratories, Hercules, CA, USA). After blotting the membranes were immersed 1 h in a blocking buffer (10 mM Tris, 100 mM NaCl, 5% non-fat dry milk, 0.2% Tween-20, pH 7.5) and incubated for 2 h at room temperature in TBS-Tween (0.2%) with an antibody against fascin-1 (1:500; sc-21743, Santa Cruz Biotechnology, Santa Cruz, CA, USA). Blots were incubated for 1 h with anti**-**mouse horseradish peroxidase–conjugated secondary antibody (1:5000; #7076, Cell Signaling Technology, Cambridge, UK) and developed using SuperSignal™ West Pico Chemiluminescent Substrate (Thermo Fisher Scientific, Waltham, MA, USA) and finally exposed with ChemiDoc™ MP System (Bio-Rad Laboratories). Protein analysis was done with the Delta2D proteome software (Decodon, Greifswald, Germany).

### Liquid chromatography-mass spectrometry (LC-MS)

3 µg protein extract of stretched and unstretched cells were reduced in 20 mM ammoniumbicarbonate by 2.5 mM dithiothreitol (DTT) for 1 h at 60 °C and with 10 mM iodoacetic acid (IAA) for 30 min at 37 °C. Subsequently the probes were digested by trypsin over night at 37 °C. MS-data were acquired on a linear-trap quadrupole Orbitrap Velos mass spectrometer (Thermo Fisher Scientific) equipped with a nanoelectrospray ion source. The resulting data were analyzed using the Proteome Discoverer 2.0 software (Thermo Fisher Scientific). SEQUEST-HT and MASCOT search engines were used for the peptide/protein identification. The database searches used the Uniprot Swiss-Prot database (version: October 2015).

### Plasmid and siRNA transfection

For plasmid transfection we used pEGFP-fascin-1-S39A (nonphosphorylatable mutant) and pEGFP-fascin-1-S39D (phosphomimetic mutant) that were kindly provided by Dr. J. Adams (Cleveland Clinic Foundation, Cleveland, OH, USA). Cells were cultured on stretch membranes for 3 days and transfected with the respective eGFP plasmid using jetPEI^®^ (Polyplus-transfection, Illkirch, France). Knockdown of fascin-1 was achieved using Silencer^®^ Select siRNAs (Ambion^®^, Thermo Fisher Scientific). For transfection with fascin-1 siRNA (final concentration 30 nM) we used the K2^®^ Transfection System (Biontex Laboratories GmbH, Munich, Germany). The cells were stretched 12–24 h after transfection. Transfection efficiency was checked by Western Blot analysis and immunofluorescence staining for fascin-1.

### Calcium switch assay

For calcium switch assays, confluent podocytes were quickly washed with PBS and subjected to EGTA (2 mM in PBS) at 38 °C. After 30 min cells were switched to culture medium (RPMI 1640, Lonza, Basel, Switzerland) and fixed with ice-cold acetone/methanol (50/50) at indicated times of recovery. For stretch experiments, cells were transfected with plasmids encoding for S39A-, S39D- or wildtype-fascin-1 48 h before EGTA treatment. After switch to RPMI 1640 mechanical stress of 7% linear strain was applied. Cells were fixed 0, 15, 60 and 90 min after stretch and visualized by GFP fluorescence of S39A-, S39D- or wildtype-fascin-1 transfected cells and by immunostaining with a rabbit anti-actin antibody (sc-1616, Santa Cruz Biotechnology).

### Immunocytochemistry

For filopodia visualisation GFP-transfected cells were fixed with acetone/methanol (50/50) for 20 min at −20 °C, washed and subjected to blocking solution (PBS, 2% fetal bovine serum, 2% bovine serum fraction V, 0.2% fish gelatine) for 1 h at room temperature. Primary antibodies were incubated for 90 min at room temperature. The following antibodies were used: mouse anti-fascin-1 (sc-21743, Santa Cruz Biotechnology) and rabbit or goat anti-actin (sc-1616, Santa Cruz Biotechnology). After a washing step with PBS (3 × 5 min) cells were incubated with secondary antibodies for 60 min at room temperature. Bound antibodies were visualized with Cy2- or Cy3-conjugated secondary antibodies (Jackson ImmunoResearch Laboratories, West Grove, USA). For nuclei staining DAPI (Sigma-Aldrich, 1:150) was used for 5 min. All samples were mounted in Mowiol (Carl Roth, Karlsruhe, Germany) and used for laser scanning microscopy (LSM) and structured illumination microscopy (SIM). Quantification of actin and fascin-1 fluorescence was achieved by measurement of fluorescence intensities of 5 stress fibers of 15 representative stretched cells from the actin rich-center (ARC) to the periphery of the cell (SigmaScanPro, Systat Software GmbH., Erkrath, Germany). For quantification of focal adhesions size and number we developed the custom software “Focal Contact Segmentation and Analysis Tool”. The focal contacts (FC) are segmented by a gradient-based local-threshold method. The specificity of this segmentation results is further increased by relating the peripheral region of each FC to its center intensity. Slightly connected FCs are separated. Finally, specific shape parameters and the fluorescence activities of each FC are computed. Additionally, the cells are segmented by a semi-automatic technique and therefore the exact position of each FC within the cell is known.

### Histology

For paraffin sections of mouse kidneys and human biopsies, samples were dehydrated and embedded in paraffin by standard procedures. Paraffin sections (5 µm) were cut on a Leica SM 2000R (Leica Microsystems, Wetzlar, Germany). After rehydration, sections were unmasked in citrate buffer (0.1 M, pH 6.0) by heating for 5 min in a pressure cooker. The sections were stained with 1 mg/100 ml Hoechst 33342 (Sigma-Aldrich) for 30 min. For immunofluorescence double-staining, samples were incubated with an antibody against nephrin (guinea pig; Progen Biotechnik GmbH, Heidelberg, Germany) and fascin-1 (HPA005723, Sigma-Aldrich,) overnight. For phospho-fascin-1 staining we used a mouse anti-fascin-1 Ser-39-phospho-specific antibody (FM3151; ECM Biosciences). Samples were washed with 1x PBS for 3 × 5 min and incubated with Cy3- and Cy2-conjugated anti-mouse/rabbit secondary antibodies (Jackson ImmunoResearch Laboratories) for 1 h. After additional washing, the samples were mounted in Mowiol (Carl Roth) for fluorescence microscopy.

### Histology on human kidney biopsies

Kidney biopsies were received from the Department of Nephropathology, Institute of Pathology, University Hospital Erlangen, Germany. The use of remnant kidney biopsy material was approved by the Ethics Committee of the Friedrich-Alexander-University of Erlangen-Nürnberg, waiving the need for retrospective consent for the use of archived rest material.

### Microarrays on human kidney biopsies

Human kidney biopsies were collected in a multicenter study (European Renal cDNA Bank-Kroener-Fresenius Biopsy Bank, ERCB^35^; for participating centers) and were obtained from patients after informed consent and with approval of the local ethics committees. Following renal biopsy, the tissue was transferred to RNase inhibitor and microdissected into glomerular and tubular fragments. Total RNA isolation from microdissected glomeruli, reverse transcription and real-time RT-PCR were performed as reported earlier^36^. Pre-developed TaqMan reagents were used for human FSCN1 (NM_003088.2), as well as the reference genes, 18 S rRNA and GAPDH (Applied Biosystems). The expression of the candidate gene was normalized to the reference genes. The mRNA expression was analyzed by standard curve quantification.

### Electron microscopy

Immunoelectron microscopy was performed on 90 nm sections of kidney tissues fixed in 2.5% paraformaldehyde in PBS and embedded in LR White (Sigma Aldrich) according to manufacturer’s protocol. After blocking in normal goat serum (Dianova) in PBST 1/30 and 0.1% BSA for 75 min fascin-1 monoclonal antibody (sc-21743, Santa Cruz Biotechnology) was applied overnight at 4 °C. After five washes in PBST, samples were incubated 1 h with secondary antibody, conjugated with gold particles of 20 nm diameter (BBInternational, Cardiff, UK). After five washings in PBST to remove unbound antibodies silver enhancement (Nanoprobes;Stony Brooks, New York, USA) was applied (5–15 min) followed by distilled water (4x). Electron micrographs were taken using an electron microscope (JEOL JEM 1011, Jeol, Peabody, MA, USA).

### Live cell microscopy

Time-lapse microscopy was done as reported previously^37^. Briefly, a custom-built Plexiglas chamber was filled with 400 µl observation medium (RPMI 1640 w/o with phenol red and 10% FBS). Cells were grown on collagen IV (0.1 mg/ml; BD Bioscience, Heidelberg, Germany) coated cover slips attached to the bottom of the chamber. The chamber was sealed and mounted on the stage of an inverted microscope. Temperature of chamber and objective was kept at 37 °C with an airstream incubator (ASI 400, Nevtek, Burnsville, VA). Images were acquired with a widefield epifluorescence microscope (Leica Microsystems, DMI6000 B, Wetzlar, Germany) equipped with Openlab software (Improvision). Images were collected in 30 s intervals over a period of 45 min.

### Laser scanning microscopy and structured illumination microscopy

Images were captured either with a Leica TCS SP5 confocal microscope (Leica Microsystems, Wetzlar, Germany), 20x/40x/63x oil immersion objectives in the Leica Application Suite software (Leica Microsystems, Version 2.6.0) or with a structured illumination microscope (SIM; ELYRA, Carl Zeiss Microscopy GmbH, Jena, Germany), 63x oil immersion objective.

### Statistical analysis

All data are given as means ± SD or ± SEM, analyzed by unpaired *t* test with repeated measurements (n). Differences were determined significant at a *p*-value < 0.05. Comparison of groups (human biopsies) was performed using Mann-Whitney *U* test.

All methods were performed in accordance with the relevant guidelines and regulations.

## Electronic supplementary material

Supplementary information

Supplementary Movie 1
