# Supplementary material for: Studying the role of fascin-1 in mechanically stressed podocytes
Source: Sci Rep. 2017 Aug 30;7:9916. doi: 10.1038/s41598-017-10116-4 (PMC5577297; doi:10.1038/s41598-017-10116-4)
Supplement: Supplementary file 1 — Supplementary information [file 41598_2017_10116_MOESM1_ESM.pdf]

Studying the role of fascin-1 in mechanical stressed podocytes

Felix Kliewe[1], Christian Scharf[2], Henrik Rogge[1], Katrin Darm[2], Maja T. Lindenmeyer[3], Kerstin Amann[4], Clemens D. Cohen[3], Karlhans Endlich[1], Nicole Endlich[1]*

[1]Department of Anatomy and Cell Biology, University Medicine Greifswald, Greifswald, Germany
[2]Department of Ear, Nose and Throat Diseases; University Medicine Greifswald, Greifswald, Germany
[3]Nephrological Center, Medical Clinic and Policlinic IV, University of Munich, Munich, Germany
[4]Department of Nephropathology; University Medicine Erlangen, Erlangen, Germany

Supplementary information

Supplementary Figures

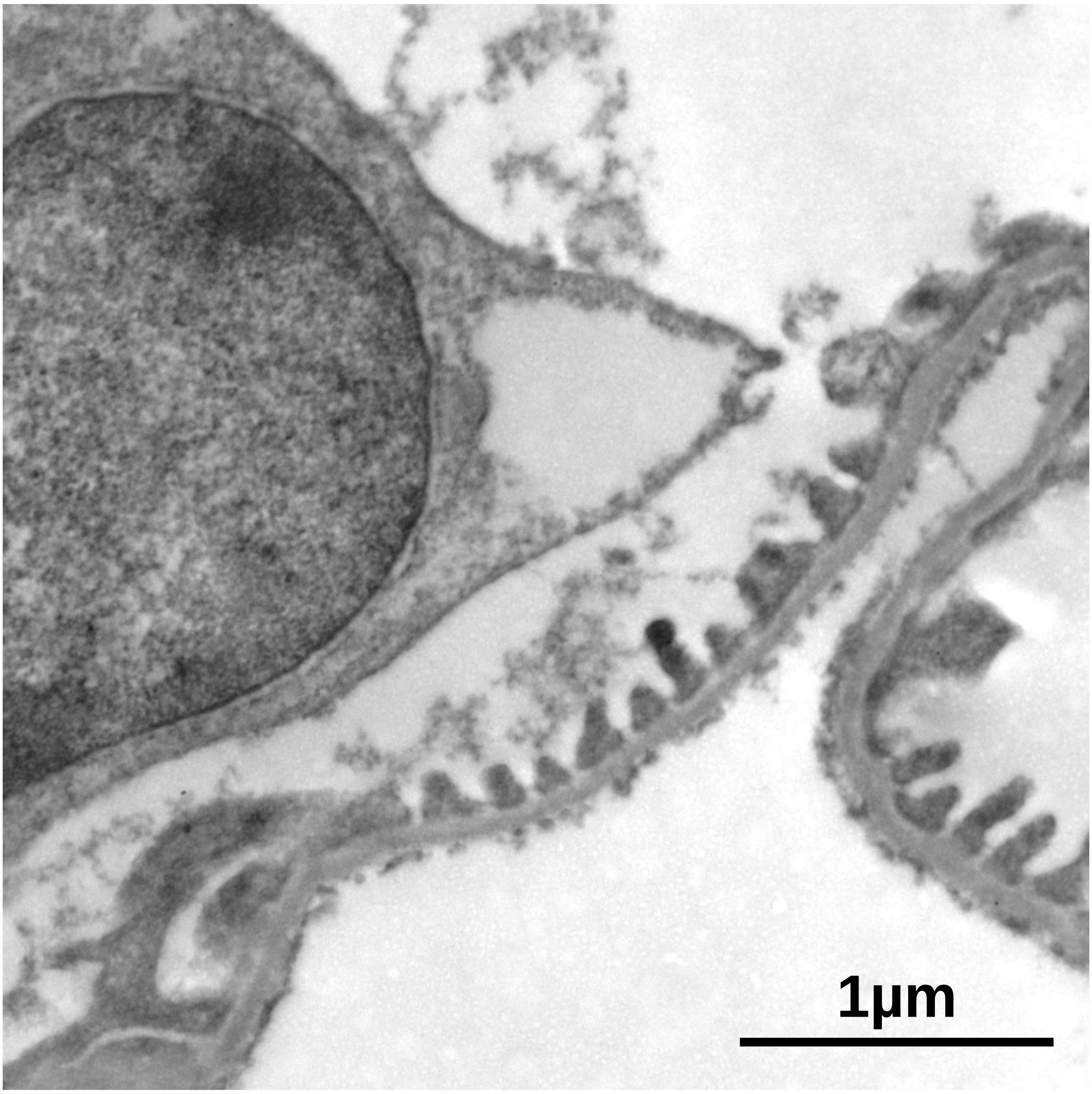

Supplementary Figure 1

Immunogold labelling: Negative control (only anti-mouse gold-labeled secondary antibody) showed no signal in podocyte cell bodies and foot processes demonstrated by EM. Scale bar represent 1 µm.

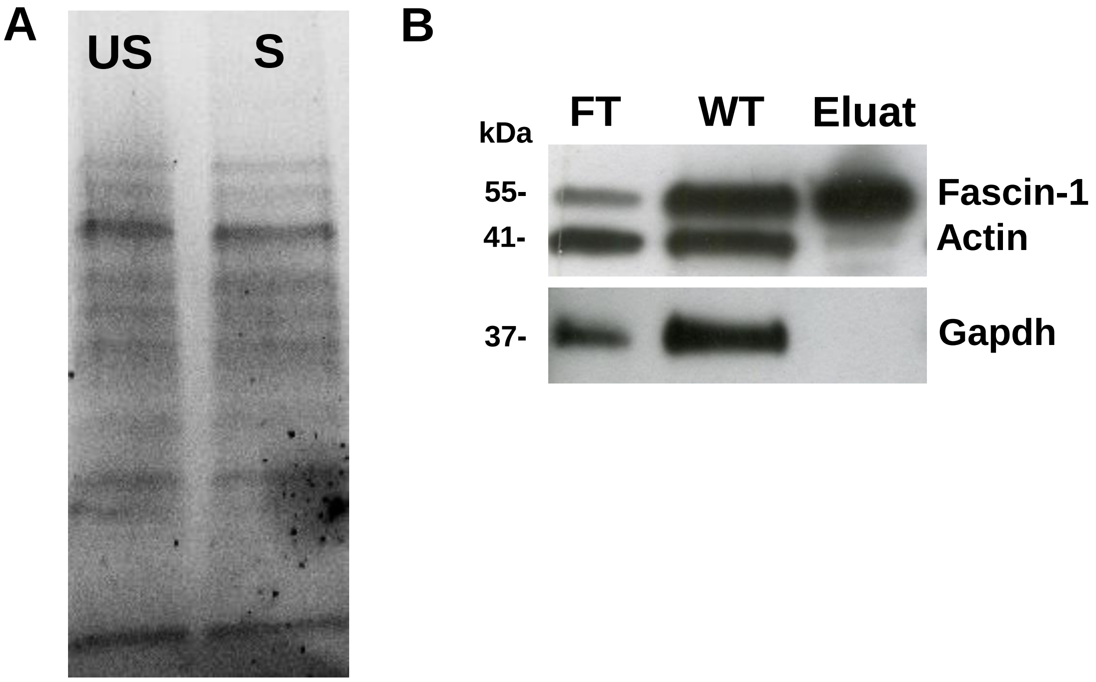

Supplementary Figure 2

(A) Protein loading from US/S phospho-enriched protein extracts was checked by stain-free imaging. (B) Phosphopeptide isolation showed an enrichment of fascin-1 in the phospho-isolated protein eluat. The housekeeping proteins like Gapdh and Actin were detect in flow through (FT) and in the wash through (WT) fractions only.

Supplementary Movies

Supplementary Movie 1:

Podocytes that were transfected with the eGFP-fascin-1-S39A plasmid spontaneously developed a large number of highly dynamic filopodia as shown by time lapse microscopy. Pictures were taken every 30 s for 45 min.
